# Determination of position and shape of flexible mri surface coils using the Microsoft Kinect for attenuation correction in PET/MRI

**DOI:** 10.1186/2197-7364-2-S1-A79

**Published:** 2015-05-18

**Authors:** Lynn Frohwein, Mirco He, Florian Buther, Klaus Safers

**Affiliations:** European Institute for Molecular Imaging, University of Muenster, Germany

Due to the varying position and shape of flexible MRI RF surface coils, the creation of attenuation maps for these coils is a challenging task. Nevertheless, coil material (metal, plastic, rubber) attenuates the PET signal to a considerable amount. Thus, including a coil μ-map into the human μ-map is essential. In this work, we present a method to determine the position and shape of flexible coils with the help of the Microsoft Kinect depth camera. Phantom PET/MRI (Siemens Biograph mMR) and CT scans (Siemens Biograph mCT) were performed with and without the flexible 32-channel coil equipped with 15 markers visible in CT and Kinect. Prior to the PET/MRI acquisition, Kinect data is acquired of the phantom with the coil on top. The manually extracted marker positions from CT and Kinect are used to non-rigidly transform the template CT according to the Kinect marker positions describing the shape of the coil during PET/MRI acquisition. An appropriate μ-map can then be calculated from the transformed CT dataset. Subsequently, the μ-map is placed in relation to the patient table according to the Kinect-derived marker positions. First results show that the coil shape can be determined with the help of the Kinect camera. The transformation of the template CT dataset according to Kinect marker positions during PET/MRI leads to appropriate results. Furthermore, the position of the coil can also be determined for an accurate placement of the μ-map in relation to the patient table. The determination of position and shape of flexible surface coils using the Kinect camera can be a way to include the CT-based coil μ-map in PET/MRI acquisitions without the need for additional MRI scans. Accuracy and practicability of the method have to be tested in further experiments.

