# Erratum to: Determination of position and shape of flexible MRI surface coils using the Microsoft Kinect for attenuation correction in PET/MRI

**DOI:** 10.1186/s40658-015-0130-3

**Published:** 2015-11-09

**Authors:** Lynn J. Frohwein, Mirco Heß, Florian Büther, Klaus P. Schäfers

**Affiliations:** European Institute for Molecular Imaging, University of Muenster, Münster, Germany

## Erratum

Unfortunately, the original version of this article [1] contained an error. The author list was included incorrectly. This can be found correctly listed above.
